# Dupilumab-Responsive Grover Disease with Concomitant Eczematous Dermatitis: A Case Report and Review of 27 Cases

**DOI:** 10.1159/cde/abvag019

**Published:** 2026-09-25

**Authors:** Sarah L. Spaulding, Jason R. McFadden, Jeffrey M. Cohen, Caroline A. Nelson, William Damsky

**Affiliations:** a Yale School of Medicine, New Haven, CT, USA; b Department of Dermatology, Yale School of Medicine, New Haven, CT, USA; c Department of Biomedical Informatics and Data Science, Yale School of Medicine, New Haven, CT, USA; d Department of Pathology, Yale School of Medicine, New Haven, CT, USA

**Keywords:** Grover disease, Transient acantholytic dermatosis, Atopy, Dupilumab, Eczematous dermatitis

## Abstract

**Introduction:**

Grover disease (GD) is a pruritic eruption with no reliably effective treatment. Emerging evidence suggests that type 2 (Th2) inflammation may contribute to GD pathogenesis. Accordingly, dupilumab has emerged as a potential therapy for recalcitrant GD. The clinical features that correlate with response remain incompletely defined; however, atopic history has been proposed as one potential marker.

**Case Presentation:**

A 68-year-old man with biopsy-confirmed GD and concomitant late-onset eczematous dermatitis, but no prior atopic history, presented with severe truncal pruritus refractory to topical and antihistamine therapies. After starting dupilumab, pruritus improved within 1 day, and his GD lesions had fully resolved by 3 months.

**Conclusion:**

Our case expands the spectrum of patients with GD who may benefit from dupilumab, lends further support for the role of Th2 cytokines in GD pathogenesis, and highlights eczematous inflammation as a feature warranting further study. Prospective studies incorporating clinical features and tissue biomarkers are needed to better identify patients with GD who are most likely to benefit from dupilumab.

## Introduction

Grover disease (GD), or transient acantholytic dermatosis, is a pruritic eruption that most commonly affects the trunk of older White men. Although GD is sometimes self-limited, other patients experience recurrent or persistent disease [1]. GD is characterized by erythematous papules, which are often eroded, and are most commonly located on photo-damaged truncal skin. GD is often accompanied by severe pruritus. Histopathology shows acantholysis with or without dyskeratosis and an inflammatory infiltrate [1]. The pathogenesis of GD remains incompletely understood, but emerging data suggest that somatic mutations in *ATP2A2*, a type 2 (Th2) polarized immune response, and MAPK pathway activation may contribute [2–4].

There remains no reliably effective or FDA-approved treatment for GD, which is often initially treated with topical steroids. Systemic retinoids, phototherapy, and immunomodulatory medications may be utilized in recalcitrant cases, but outcomes are variable and often suboptimal [1].

It has been observed that upregulation of interleukin-13 (IL-13) occurs in lesional skin in most patients with GD [5]. Consistent with this, several reports have demonstrated that patients with GD can respond to dupilumab [6–8]. Clinical features that identify potentially dupilumab-responsive patients remain incompletely defined; however, a recent case series of 10 patients found that those with a history of atopy may be more likely to respond [3]. We present a patient with GD with late-onset eczematous dermatitis, but not longstanding atopy, who demonstrated a robust response to dupilumab, adding to growing evidence that coexisting Th2 inflammation may correlate with response to dupilumab in patients with GD.

## Case Report/Case Presentation

A 68-year-old male with a history of prostate cancer presented to our clinic with a 6-month history of GD and an eczematous eruption that the patient reported noting around the same time. Three biopsies had been performed: two from crusted papules on the trunk, both showing acantholytic dyskeratosis (consistent with GD), and one from an eczematous plaque on the right posterior thigh showing spongiotic dermatitis without acantholysis (consistent with eczematous dermatitis) (Fig. 1a, b). There were no new medications, and he had no history of prior atopic disease. The patient had been previously unsuccessfully treated with hydroxyzine, diphenhydramine, halobetasol 0.05% cream, and tacrolimus 0.1% ointment.

**Fig. 1.**
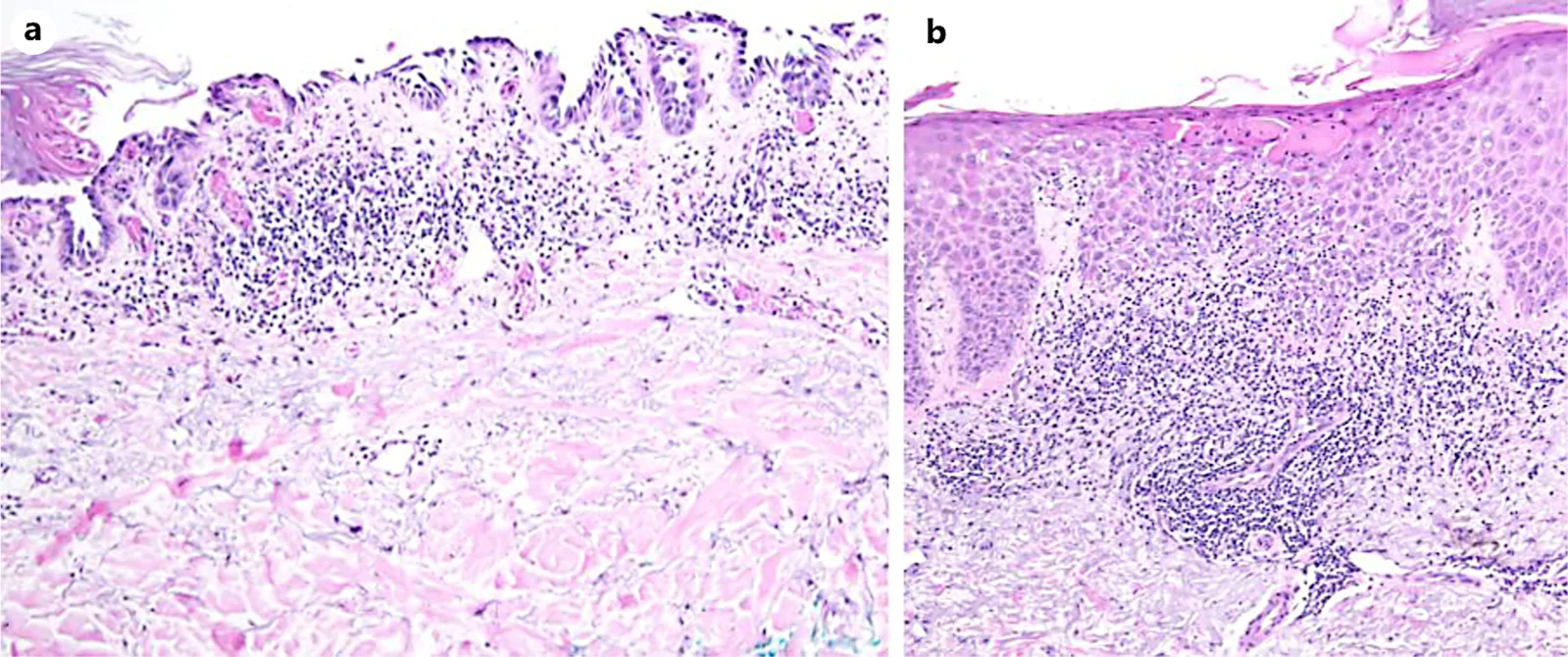
Representative histopathologic images. **a** Biopsy from the trunk showing suprabasilar acantholysis, focal dyskeratosis, and an inflammatory infiltrate with occasional eosinophils, H&E, ×100. **b** Biopsy from the right posterior thigh showing spongiotic dermatitis without acantholysis, H&E, ×100.

On examination, there were widespread pink papules, many centrally eroded, on the chest, abdomen, flanks, and back (shown in Fig. 2a, b). Peak pruritus numerical rating scale (PP-NRS) was 9/10. Due to the widespread distribution of the GD and the severe pruritus, the patient was initiated on dupilumab (a 600 mg loading dose, followed by 300 mg every 14 days). PP-NRS improved from 9/10 to 1/10 after 1 day of treatment. No concomitant medications for GD were continued after dupilumab initiation; all had been discontinued before the first dose. At the 3-month follow-up visit, the GD lesions had resolved (Fig. 2c, d), and the eczematous lesions had greatly improved. PP-NRS was 0. The patient’s GD has remained well-controlled for over 6 months on dupilumab.

**Fig. 2.**
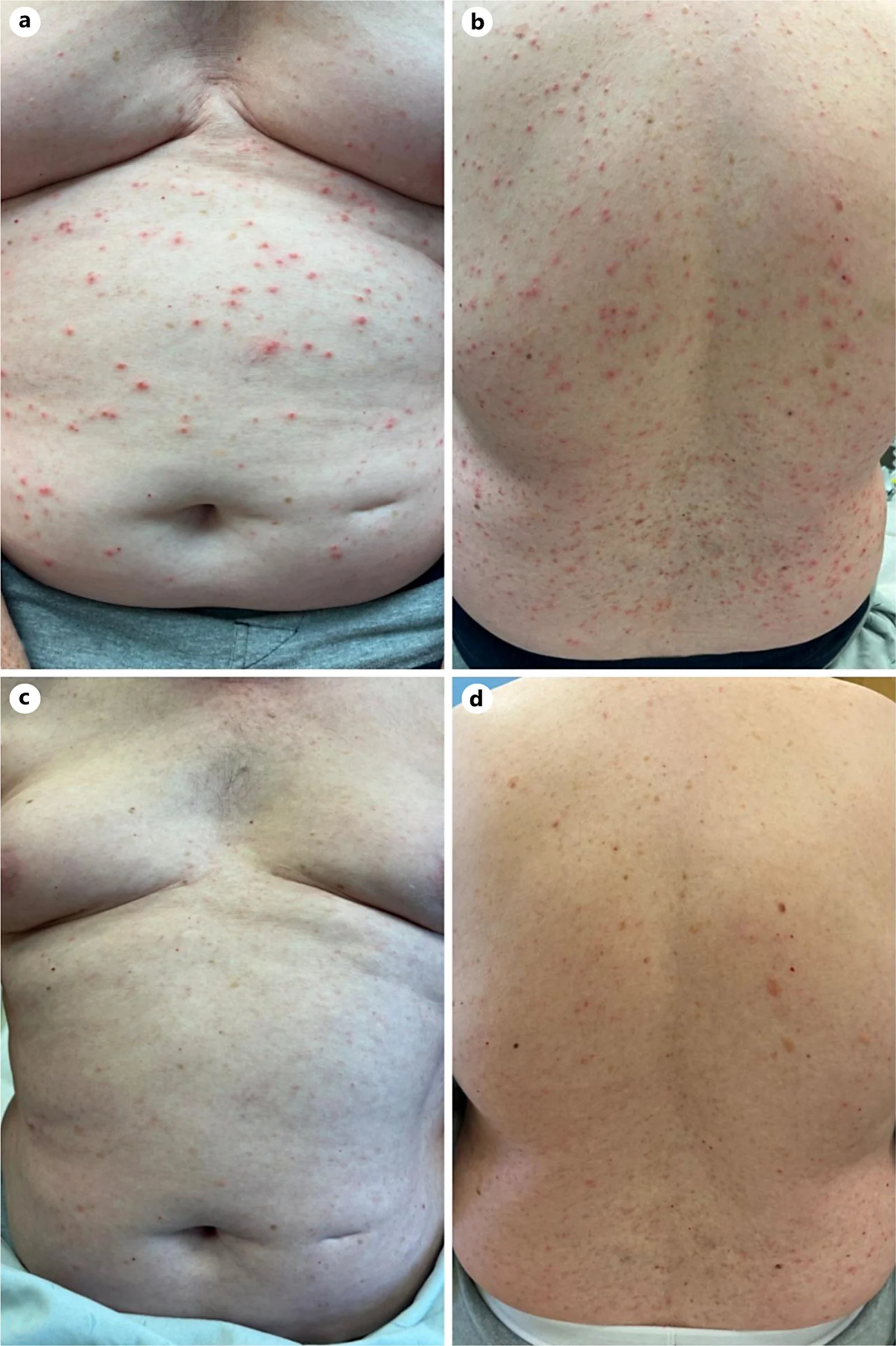
GD (transient acantholytic dermatosis). **a**, **b** Diffuse, scattered erythematous papules before dupilumab. **c**, **d** Clearance of the eruption is noted 3 months after initiation of dupilumab.

## Discussion

The emergence of dupilumab and other Th2-targeted biologics has introduced new therapeutic possibilities for GD. Whereas prior reports have suggested a history of atopy as a predictor of response to dupilumab in GD, this case suggests that patients without a history of atopy, but with concomitant eczematous dermatitis, may also respond well to dupilumab.

In order to summarize and evaluate the evidence of Th2-targeted therapies in GD, we performed a structured literature review for publications using the terms “Grover disease” or “Grover’s disease” or “transient acantholytic dermatosis” and “dupilumab,” “lebrikizumab,” “tralokinumab,” or “nemolizumab.” Seven publications comprising 27 patients with GD treated with standard AD dosing of dupilumab were identified (shown in Table 1). No patients treated with other Th2-targeted therapies were found.

**Table 1.** Summary of prior published reports of dupilumab use in GD

	Study	Patients^a^, *n*	Complete dupilumab response, *n* (%)	Partial dupilumab response^b^, *n* (%)	Mean age at biopsy (range), years	Hx of atopy^c^, *n* (%)	Male sex, *n* (%)
1	Butler et al., 2021 [6]	3	3 (100)	0 (0)	NR (approx. 50–79)	NR	2 (67)
2	Barei et al., 2022 [7]	1	1 (100)	0 (0)	77	NR	1 (100)
3	Kaprive et al., 2024 [8]	1	1 (100)	0 (0)	70	NR	0 (0)
4	Amara et al., 2025 [9]	10	9 (90)	1 (10)	70.3 (59–81)	7 (70)	7 (70)
5	Murphy et al., 2025 [5]	9	5 (56)	4 (44)	NR (55–78)	3 (33)^d^	8 (89)
6	Magnano et al., 2025 [9]	2	2 (100)	0 (0)	63.5 (54–73)	1 (50)	2 (100)
7	McCarter et al., 2025 [10]	1	1 (100)	0 (0)	73	NR	1 (100)
Total		27	22 (81)	5 (19)	–	11 (41)	21 (78)

NR, not recorded.

^a^ With GD treated with ≥1 dose of dupilumab.

^b^ Any improvement in lesions and/or pruritus that did not meet complete response criteria.

^c^ Current or prior atopic disease (atopic dermatitis, hay fever, seasonal allergies, and/or asthma).

^d^ Available atopic history was limited to concomitant atopic dermatitis.

Twenty-two patients (81%) achieved a complete response to dupilumab, which we defined as complete lesion clearance (0% BSA or author-stated clearance) and/or pruritus resolution (final PP-NRS score = 0 or author-stated resolution) at follow-up. The remaining 5 (19%) had a partial response. Itch reduction tended to be observed within days to weeks, and lesion reduction was observed over weeks to months.

Although most patients did well with dupilumab, analysis of case reports may be biased towards the best responders. Therefore, identifying clinical features that might correlate with responsiveness to dupilumab in GD would be useful. In a retrospective study of 10 patients with GD treated with dupilumab, Amara and colleagues found that 7 patients with prior or current atopy (AD, asthma, or seasonal allergies) achieved complete response, compared to 2/3 (67%) without atopy [3]. Across other published dupilumab-treated GD cases, 4 had an atopic history, 7 did not have atopy, and 6 did not specify, suggesting that lack of an atopic history does not preclude response.

Ultimately, whether atopy is associated with a higher likelihood of response remains uncertain. Our current case expands this framework, suggesting that concurrent eczematous dermatitis, even without a history of atopy, may also be relevant.

Tissue biomarkers may also be of interest in predicting responsiveness to dupilumab in GD. In a published series [5], we previously used RNA in situ hybridization staining to show that there was a significant increase in *IL13* mRNA expression in skin biopsies of GD compared to healthy controls. Levels of *IL13* rivaled those seen in AD in some patients and were detectable in 25/31 (81%) of cases [5]. Interestingly, we found that the absence of *IL13* staining did not necessarily preclude response to dupilumab: 2 patients without detectable *IL13* staining responded [5]. Moving forward, more comprehensively evaluating tissue biomarkers (e.g., *IL13* staining), other histologic features (e.g., degree of background spongiosis or eosinophils), other clinical features (e.g., degree of pruritus), and other laboratory findings (e.g., serum IL-13 levels, IgE levels, and eosinophilia) and response to dupilumab will also be of great interest.

Strengths of this analysis include biopsy confirmation of both GD and concomitant eczematous dermatitis, the longitudinal follow-up showing sustained disease control for more than 6 months, and the contextualization of our case within the growing body of published experience with dupilumab-treated GD. Regarding limitations, the presence of concomitant eczematous dermatitis makes it difficult to determine the extent to which dupilumab directly improved GD versus treating an overlapping eczematous or Th2-driven inflammatory process. Additionally, the literature review is limited by the small number of published cases, variable follow-up durations, and likely publication bias favoring successful responses.

Dupilumab has emerged as a promising therapy for patients with severe or recalcitrant GD. Our case suggests that, in addition to a history of atopy, nonatopic eczematous dermatitis may be associated with clinical response to dupilumab in GD. It also lends further support for the role of Th2 cytokines in GD pathogenesis. However, this observation is based on a single case and should be considered hypothesis-generating. Further research is needed to clarify which subsets of patients with GD are most likely to benefit from dupilumab, and likely will only occur through well-designed prospective, randomized controlled studies. The CARE checklist was used in preparing this manuscript and is available as online supplementary material (for all online suppl. material, see https://doi.org/10.1159/cde/abvag019).

## Statement of Ethics

Ethics approval was not required for this manuscript, per the following guidance from the Yale University Institutional Review Board (IRB): “Case studies do not meet the Common Rule definition of research because it is not a systematic investigation designed to develop or contribute to generalizable knowledge. As case reports, by definition, have a very limited sample size, they are not designed to be predictive of similar circumstances and hence do not meet the generalizable requirement of this definition and therefore are not subject to IRB review.” The authors obtained written informed consent from the patient for their photographs and medical information to be published in print and online and with the understanding that this information may be publicly available. Patient consent forms were not provided to the journal but are retained by the authors.

## Conflict of Interest Statement

W.D. is a consultant for Pfizer, Incyte, Eli Lilly, and TWi Biotechnology, has research funding from Pfizer, Advanced Cell Diagnostics/Bio-techne, and AbbVie and receives licensing fees from EMD/Millipore/Sigma, all outside the submitted work. J.M.C. serves on a data and safety monitoring board (DSMB) for Advarra and has served as a consultant for Novartis, Takeda, GSK, and Sanofi. C.A.N. received an unrelated grant from and served on an advisory board for Boehringer Ingelheim. S.L.S. and J.R.M. have no conflicts of interest to disclose.

## Funding Sources

This study was not supported by any sponsor or funder.

## Author Contributions

S.L.S. and J.R.M. contributed to the literature review, data collection, data analysis, drafting of the manuscript, and made equal contributions to this study. J.M.C. contributed to data interpretation and critical revision of the manuscript. C.A.N. contributed to data interpretation and critical revision of the manuscript. W.D. contributed to clinical care of the patient, study conception and supervision, data interpretation, and critical revision of the manuscript. All authors reviewed and approved the final version of the manuscript.

## Data Availability

The data supporting the findings of this case report and literature review, including previously published case reports and case series, are included within the article. Deidentified data from our prior cytokine expression analyses using RNA in situ hybridization (RISH) are available from the corresponding author upon request. Additional patient-level clinical data are not publicly available.
